# Joint effects of depression and social determinants of health on mortality risk among U.S. adults: a cohort study

**DOI:** 10.1186/s12888-024-06159-3

**Published:** 2024-10-30

**Authors:** Zun Wang, Boxuan Pu

**Affiliations:** 1 Youanmen Community Healthcare Center of Fengtai District, Beijing, China; 2 National Clinical Research Center for Cardiovascular Diseases, Fuwai Hospital, National Center for Cardiovascular Diseases, Chinese Academy of Medical Sciences and Peking Union Medical College, Beijing, China

**Keywords:** Depression, Social determinants of health, Joint effects, Mortality, Adults, NHANES

## Abstract

**Background:**

Unfavorable social determinants of health (SDoH) are associated with depression. Both depression and SDoH are associated with increased risks of mortality, but their joint impacts on mortality risks remain unclear. This study aims to investigate the joint effects of depression and SDoH on mortality risk.

**Methods:**

Utilizing data from the National Health and Nutrition Examination Survey (NHANES) 2007–2018, 24,727 adults aged ≥ 20 were included. SDoH was assessed based on the 5 domains outlined in the U.S. Healthy People 2030 initiative. The cumulative number of unfavorable SDoH was calculated and categorized into low and high burden levels. The definition of depression was based on the Patient Health Questionnaire-9 (PHQ-9) scores ≥ 10. The joint associations of depression and SDoH with all-cause, cardiovascular disease (CVD), and cancer mortality were examined using Cox proportional hazard models.

**Results:**

We identified 2,377 (6.84%) all-cause deaths (CVD, 717; cancer, 606) during a median follow-up of 7.0 years. Depression was associated with increased mortality risks, and SDoH could explain 32.4% and 28.3% of the associations between depression and all-cause and CVD mortality, respectively. No significant interactions were observed between depression and SDoH on mortality. However, a low burden of unfavorable SDoH reduced the risk of all-cause mortality in depressed patients (hazard ratio [HR], 0.58; 95% confidence interval [CI]: 0.36–0.92). In the joint analysis, individuals with both depression and a high burden of unfavorable SDoH had the highest risks of all-cause and CVD mortality. Specifically, compared with individuals with no depression and a low burden of unfavorable SDoH, those with depression and a high burden of unfavorable SDoH had higher risks of all-cause (HR, 2.52; 95% CI: 2.01–3.18) and CVD mortality (HR, 2.79; 95% CI: 1.95–3.99).

**Conclusion:**

Adults with both depression and a high burden of unfavorable SDoH had the highest risks of all-cause mortality and CVD mortality. The result suggests considering depression and SDoH jointly in developing targeted intervention strategies to improve survival outcomes and calls for larger cohort studies and clinical trials to validate our findings.

**Supplementary Information:**

The online version contains supplementary material available at 10.1186/s12888-024-06159-3.

## Introduction

Depression is a common mental disorder that severely limits psychosocial functioning and diminishes quality of life [1]. It has emerged as the leading cause of mental disease burden and disability, affecting more than 350 million people worldwide [2]. According to the World Health Organization (WHO), depression significantly contributes to a substantial disease burden worldwide, as underscored as the third contributor to global disease burden in 2008, with a projected progression to the foremost leading position by 2030 [3]. Depression itself can disrupt immunological, endocrine, and neurological functions, thereby increasing vulnerability to various diseases, such as cardiovascular diseases (CVD) and cancer, and even result in death [4]. Association of depression with mortality has been examined systematically with several hundred studies and abundant studies suggested that depression was associated with increased risk of all-cause and CVD mortality [5, 6], which underscores the need for a comprehensive strategy to reduce the mortality risk due to depression.

The associations of residence, marital status, education, and income with depression were demonstrated drawing to the attention of the importance of social determinants of health (SDoH) [7, 8]. SDoH have been recognized as contributing and modifiable risk factors to disparities in depressive outcomes [9, 10]. The WHO defines SDoH as “the conditions in which people are born, grow, live, work, and age, and the wider set of forces and systems shaping the condition of daily life” [11]. The U.S. Healthy People 2030 offered a comprehensive framework for SDoH across five key domains: economic stability, education access and quality, health care access and quality, neighborhood and built environment, and social community context [12]. In epidemiological studies, unfavorable SDoH have been associated with adverse clinical outcomes, such as elevated prevalence of CVD, cancer, and premature mortality [13–15]. Furthermore, previous research has demonstrated a bidirectional relationship between depression and SDoH. Individuals who face multiple unfavorable SDoH not only face more stress, adversity, and social challenges but also lack the personal resources and social support to cope with these stressful events [7, 16]. Consequently, they are more likely to suffer from depression and become more vulnerable to adverse events [17, 18]. Simultaneously, depression may lead to adverse SDoH metrics [7, 19]. Additionally, the relationship between depression and mortality risks was found to be stronger among individuals with low socioeconomic status (SES), which is the commonly studied SDoH [20]. Despite growing evidence on the importance of these two risk factors, some knowledge gaps emerge. First, whether SDoH mediates the relationship between depression and mortality remains unknown. Second, it is unclear how SDoH may interact with depression on mortality. Additionally, a limited number of previous studies reported that the co-existence of depression and low SES was related to increased risks of adverse clinical outcomes [21, 22]. However, the joint association of depression and SDoH with mortality in the general population have rarely been studied. Given the potential interplay between depression and SDoH as well as their impact on mortality, it is crucial to enhance our understanding of their joint effects on mortality risks for effective public health strategies to prevent mortality.

Accordingly, we used data from the National Health and Nutrition Examination Survey (NHANES) 2007–2018. This study included a large, nationwide representative sample of the U.S. population. By leveraging these data, we aimed to investigate (1) whether SDoH mediates the association between depression and mortality, (2) the interaction between depression and SDoH on mortality, and (3) the joint effects of depression and SDoH on mortality risks.

## Methods

### Study design and population

This cohort study included a nationally representative sample of the civilian noninstitutionalized U.S. population from NHANES, which is an ongoing biannual series of surveys that collects health and nutrition-related data and uses a complex, stratified, multistage probability sampling design. Participants were selected through a four-stage probability sampling design with primary sampling units selected at the county level, census trace level, household level, and individual level in the 50 states and the District of Columbia. Considering the complicated survey design, including oversampling, survey nonresponse, and poststratification, sample weights are provided. Following a home interview, participants were invited to a mobile screening facility to undergo anthropometric measurements, physiological examinations, and blood testing. The National Center for Health Statistics Research Ethics Review Board approved the NHANES protocols, and each participant provided signed written informed consent. This study was based on the data collected from 2007 to 2018 across 6 cycles. Eligible participants included adults aged ≥ 20 years old with available information on depression, SDoH, and morality. Exclusion criteria were including (1) individuals aged < 20 years (*n* = 25,072), considering the definition of adults in most studies using NHANES data [13, 23], (2) pregnant women (*n* = 372), based on literature and being pregnant was associated with depression risk and more susceptible to adverse SDoH [23–25], (3) individuals without available mortality data (*n* = 115), (4) individuals without values on any individual domain for SDoH (*n* = 2,645) and Patient Health Questionnaire-9 (PHQ-9) (*n* = 4,823), (5) individuals with missing values on covariates (*n* = 2,088). The final analysis included 24,727 participants (Supplementary Figure S1).

### Assessment of SDoH

Self-reported 8 sub-items of SDoH across 5 domains were operationalized according to the criteria outlined in U.S. Healthy People 2030 initiative and previous two studies, with a cumulative measure of unfavorable SDoH calculated for analysis [12, 13, 26]. These domains include economic stability (employment status, family poverty-income ratio, and food security), education access and quality (education level), health care access and quality (health insurance coverage and type of health insurance), neighborhood and built environment (home ownership), and social and community context (marital status). The specific definitions of SDoH domains and sub-items are shown in Supplementary Table S1. To simplify analysis, we dichotomized these SDoH items into favorable or unfavorable levels based on conventional cutoff points. The cumulative number of unfavorable SDoH was calculated by summing up the 8 dichotomized SDoH items with a value of 0 representing a favorable level and a value of 1 representing an unfavorable level. According to previous studies, participants were divided into groups with a low burden of unfavorable SDoH (SDoH ≤ 2) and a high burden of unfavorable SDoH (SDoH > 2) [27, 28].

### Assessment of depression

Depression was assessed using the PHQ-9, which is a validated self-report 9-item screening instrument based on the mood module in Diagnostic and Statistical Manual of Mental Disorders-IV and has excellent reliability (Cronbach’s a > 0.85 in 2 different studies) and validity (Area under curve = 0.95) [29]. The PHQ-9 asks about the frequency of depressive symptoms during the past 2 weeks before the survey. The PHQ-9 consists of nine items on depressive symptoms (lack of interest, depressed mood, trouble sleeping, fatigue, appetite problems, worthlessness, lack of concentration, psychomotor agitation or retardation, and suicidal thoughts). The response options “not at all”, “several days”, “more than half the days”, and “nearly every day” for each item received scores ranging from 0 to 3, respectively. Thus, the total scores range from 0 to 27, with higher scores indicating more severe depressive symptoms. Individuals with a total score ≥ 10 were considered to suffer from depression, the sensitivity of this threshold was 88% and the specificity was 88% [29]. This cut-off value is also a well-validated cutoff point commonly used in most clinical studies of depression and considered to indicate clinically significant depression [30]. In this study, participants were classified into 2 categories based on PHQ-9 scores: no depression (PHQ-9 < 10), and depression (PHQ-9 ≥ 10).

### Outcomes

The primary outcome was all-cause mortality. Secondary outcomes were cause-specific mortality, including CVD mortality and cancer mortality. The participant death information was identified by the NCHS through linkage with the National Death Index until December 31, 2019. The International Classification of Diseases, Tenth Revision codes were used to identify underlying causes of mortality. CVD death was defined as death due to diseases of heart (codes I00 to I09, I11, I13, I20 to I51, I60 to 169), while cancer death referred to deaths caused by cancer (codes C00-C97). Follow-up time for each participant was calculated from the baseline examination date until either the date of death or last known survival date (December 31, 2019), whichever came first.

### Covariates

Covariates included demographic characteristics (age, sex, race/ethnicity), lifestyle factors (smoking status, drinking status, physical activity [PA], sleep duration), clinical characteristics (body mass index [BMI], CVD, cancer, hypertension, diabetes mellitus [DM], dyslipidemia). Race/ethnicity were categorized as non-Hispanic Black, non-Hispanic White, Mexican American, or other races. Smoking status was classified into three groups: current smokers (smoked ≥ 100 cigarettes during the lifetime and continue to smoke), former smokers (smoked ≥ 100 cigarettes during the lifetimes but have quit smoking), or never-smokers (smoked < 100 cigarettes during the lifetime). Drinking status was categorized into never (< 12 alcohol drinks during the lifetime), former (≥ 12 alcohol drinks during the lifetime and not drink alcohol over past 12 months), and current drinkers (had ≥ 12 alcohol drinks during the lifetime and still drink). PA was assessed using the Global Physical Activity Questionnaire by calculating the sum of minutes engaged in moderate-intensity activities plus twice the minutes spent in vigorous-intensity activities. PA level was further categorized as PA < 150 min/week and PA ≥ 150 per/week. Sleep was assessed using the self-report question “How much sleep do you get (in hours)” and categorized into < 7 h and ≥ 7 h.

During the physical examination, weight and height were measured and BMI was calculated as weight (kg) divided by height (m) squared. BMI was categorized into 3 groups (< 25, 25-29.9, ≥ 30 kg/m^2^). CVD and cancer were identified based on self-reported physician diagnoses obtained during individual interviews using standardized medical condition questionnaires. CVD included heart failure, coronary heart disease, angina pectoris, myocardial infarction, and stroke. Additionally, hypertension was identified by self-reported diagnosis, ongoing use of antihypertensive medications, or meeting any of the following criteria: systolic blood pressure/diastolic blood pressure ≥ 140/90 mmHg. DM was defined by self-reported diagnosis, current insulin or antidiabetic medication use, or meeting any of the following criteria: fasting plasma glucose levels ≥ 7.0 mmol/L, postprandial 2-h plasma glucose levels ≥ 200 mg/dL, or glycated hemoglobin A1c ≥ 6.5%. Dyslipidemia was ascertained based on self-reported diagnosis, prescribed lipid-lowering medications, or meeting any of the following criteria: total cholesterol ≥ 200 mg/dL, triglyceride ≥ 150 mg/dL, low-density lipoprotein cholesterol ≥ 130 mg/dl, or high-density lipoprotein cholesterol < 40 mg/dL for men and < 50 mg/dL for women.

### Statistical analyses

All analyses were conducted following the NHANES analytic guidelines for the sample weights, clustering, and stratification to estimate appropriate variance and ensure nationally representative of the U.S. population. Generally, in all weighted analyses, we first included a masked variance of the primary sampling unit (sdmvpsu) to allow for clustering and the unequal selection probability of counties or small groups of contiguous counties. Then, we included a pseudo-stratum masked variance (sdmvstra) to allow for the oversampling of age, ethnic, or income subgroups. Finally, we constructed individual-level sampling weights across the 6 consecutive NHANES survey cycles by using the 2-year mobile examination center weight divided by 6.

We reported weighted mean (standard error [SE]) and counts (weighted percentages) for continuous and categorical variables. We utilized analysis of variance to compare continuous variables and the chi-square test for categorical variables for the distribution of characteristics between participants with different statuses of depression or SDoH.

Hazard ratios (HRs) and 95% confidence intervals (CIs) for the individual and joint associations of depression and SDoH with all-cause, CVD, and cancer mortality were estimated using Cox proportional hazards regression models. Model 1 was unadjusted. Model 2 was adjusted for multiple covariates including age, sex, race/ethnicity, smoking status, drinking status, PA, sleep, BMI, CVD, cancer, hypertension, DM, and dyslipidemia. The mediating effect of SDoH on the association between depression and morality was determined using the R package “mediation.” To assess the proportion of mediating effects, we used the difference method by comparing estimates from multivariate models with and without the hypothesized mediator, i.e., models including depression and SDoH versus models excluding SDoH.

We further conducted a stratified analysis of the association between depression and mortality stratified by SDoH levels, with no depression as the reference group. Additionally, we included an additional product term of depression and SDoH in the multivariate Cox models to quantify both additive and multiplicative interactions. We used the relative excess risk due to interaction (RERI), attributable proportion (AP), and synergy index (SI) as the measures of additive interaction, and estimated HR for interaction terms for the multiplicative interaction. The additive interaction of RERI and AP was significant when its CI did not include 0, and the SI was significant when its CI did not include 1. Multiplicative interaction was significant when its CI did not contain 1. Furthermore, to assess the joint associations, participants were divided into four groups based on depression statuses (yes or no) and categories of SDoH (low or high burden of unfavorable SDoH), and mortality risks were estimated compared to those with a low burden of unfavorable SDoH and no depression as the reference group. Kaplan-Meier (KM) curves were also used to display the different survival probabilities among these groups.

Two sensitivity analyses were conducted to assess the robustness of the findings: (1) exclusion of participants who died within the first 2 years of follow-up, (2) exclusion of participants who had CVD or cancer at baseline. Additionally, a stratified subgroup analysis was performed among participants with or without depression to explore whether a low burden of unfavorable SDoH could decrease mortality risks from depression. Several subgroup analyses by age (< 65 years, ≥ 65 years), sex (men, women), race/ethnicity (white, nonwhite), CVD (yes, no), and cancer (yes, no) were conducted to explore potential variations across different subgroups. All analyses were conducted using R Version 4.2.2 (R Foundation for Statistical Computing, Vienna, Austria), and two-sided P values < 0.05 were considered to indicate statistical significance.

## Results

### Participant characteristics

Among the 24,727 NHANES participants (weighted population: 174,349,192), the mean (SE) age was 47.28 (0.28) years, 12,530 (50.67%) were women, and 2,244 (7.97%) had depression. Compared to those without depression, participants with depression were more likely to be women, white people, with less sleep duration, current drinkers, lack of physical activity, higher BMI, and have CVD, cancer, hypertension, DM, and dyslipidemia. Moreover, there were 14,229 (55.78%) participants with a high burden of unfavorable SDoH. Participants with a high burden of unfavorable SDoH tended to be younger, more likely to be women, nonwhite people, current smokers, with CVD, DM, higher BMI, and have less sleep duration and physical activity compared to those with a low burden of unfavorable SDoH (Table 1).

**Table 1:** Baseline characteristics of participants

Characteristics	Total (*n* = 24727)	Unfavorable SDoH			Depression		
		Low burden (*n* = 10498)	High burden (*n* = 14229)	*P* value	Yes (*n* = 2244)	No (*n* = 22483)	*P* value
Weighted N (weighted %)	174,349,192 (100.0)	77,103,552 (44.22)	97,245,640 (55.78)		13,886,966 (7.97)	160,462,226 (92.03)	
Demographic characteristics							
Age, years (SE)	47.28 (0.26)	48.99 (0.28)	45.11 (0.37)	< 0.001	46.58 (0.45)	47.34 (0.28)	0.141
Women, n (weighted %)	12,530 (50.67)	5138 (49.90)	7392 (52.69)	< 0.001	1436 (64.41)	11,094 (49.98)	< 0.001
Race/ethnicity, n (weighted %)				< 0.001			0.002
Mexican	3583 (7.98)	928 (3.96)	2655 (13.04)		320 (7.34)	3263 (8.03)	
White	10,756 (68.75)	5626 (79.46)	5130 (55.25)		990 (65.39)	9766 (69.04)	
Black	5162 (10.67)	1765 (6.55)	3397 (15.86)		470 (12.84)	4692 (10.48)	
Other	5226 (12.60)	2179 (10.03)	3047 (15.85)		464 (14.42)	4792 (12.45)	
Lifestyle factors, n (weighted %)							
Sleep duration ≥ 7 h	16,011 (68.02)	7001 (70.29)	9010 (65.14)	< 0.001	1130 (53.09)	14,881 (69.31)	< 0.001
Drinking status				< 0.001			0.001
Never	3519 (10.82)	1178 (8.55)	2341 (13.68)		270 (8.93)	3249 (10.98)	
Former	3013 (9.96)	1183 (9.22)	1830 (10.89)		302 (11.93)	2711 (9.79)	
Current	18,195 (79.22)	8137 (82.23)	10,058 (75.43)		1672 (79.14)	16,523 (79.23)	
Smoking status				< 0.001			< 0.001
Never	13,643 (55.55)	6480 (60.72)	7163 (49.02)		900 (38.86)	12,473 (56.99)	
Former	5960 (24.74)	2777 (26.88)	3183 (22.04)		491 (21.49)	5469 (25.02)	
Current	5124 (19.71)	1241 (12.39)	3883 (28.95)		853 (39.65)	4271 (17.99)	
Physical activity ≥ 150 min/week	6512 (30.82)	3513 (36.40)	2999 (23.79)	< 0.001	302 (15.07)	6210 (32.19)	< 0.001
Clinical characteristics							
CVD, n (weighted %)	2586 (8.20)	788 (6.13)	1798 (10.81)	< 0.001	429 (15.75)	2157 (7.55)	< 0.001
Cancer, n (weighted %)	2419 (10.46)	1187 (11.82)	1232 (8.75)	< 0.001	255 (11.17)	2164 (10.40)	0.446
Hypertension, n (weighted %)	9008 (32.23)	3701 (31.81)	5307 (32.75)	0.311	1062 (43.59)	7946 (31.25)	< 0.001
Diabetes mellitus, n (weighted %)	4370 (13.26)	1562 (11.69)	2808 (15.23)	< 0.001	570 (19.66)	3800 (12.70)	< 0.001
Dyslipidemia, n (weighted %)	8383 (33.02)	3975 (37.02)	4408 (27.98)	< 0.001	915 (38.49)	7468 (32.55)	< 0.001
Body mass index, kg/m^2^ (SE)	29.16 (0.12)	28.95 (0.11)	29.41 (0.10)	0.001	30.80 (0.24)	29.01 (0.09)	< 0.001

Data are survey-weighted mean (SE) or N (weight% %)

Abbreviations: SE: standard error; SDoH: social determinants of health; CVD: cardiovascular diseases

### Mediation analysis of SDoH on associations of depression with mortality

Over a median follow-up period of 7.0 years (IQR, 4.3–10.0), there were 2,377 all-cause deaths (weighted mortality: 6.84%) occurred, including 717 (1.96%) CVD deaths and 606 (1.77%) cancer deaths. Multivariate analyses showed that participants with depression had significantly elevated risks of all-cause (HR, 1.48; 95% CI: 1.27–1.73) and CVD mortality (HR, 1.62; 95%CI: 1.22–2.15) compared to those without depression. Compared to the low burden of unfavorable SDoH group, the high burden of unfavorable SDoH group was associated with increased risks of all-cause (HR, 2.05; 95% CI: 1.82–2.31), CVD (HR, 2.09; 95% CI: 1.70–2.57) and cancer mortality (HR, 1.51; 95% CI: 1.24–1.85), respectively (Table 2).

**Table 2:** Individual association of depression and social determinants of health with all-cause and cause-specific mortality

Outcomes	Death/No	Weighted death (%)	Model 1 HR (95%CI)	*P* value	Model 2 HR (95%CI)	*P* value
**All-cause mortality**						
Depression						
No	269/2,244	1,312,518 (9.45)	1 (Reference)		1 (Reference)	
Yes	2,108/22,483	10,605,879 (6.61)	1.45 (1.23–1.70)	< 0.001	1.48 (1.27–1.73)	< 0.001
Per 1 PHQ-9 score increase	NA	NA	1.04 (1.03–1.05)	< 0.001	1.04 (1.03–1.06)	< 0.001
Unfavorable SDoH						
Low burden	697/10,498	4,485,286 (4.61)	1 (Reference)		1 (Reference)	
High burden	1,680/14,229	7,433,112 (9.64)	2.21 (1.93–2.52)	< 0.001	2.05 (1.82–2.31)	< 0.001
Per 1 unfavorable SDoH increase	NA	NA	1.24 (1.21–1.27)	< 0.001	1.26 (1.22–1.29)	< 0.001
**CVD mortality**						
Depression						
No	81/2,244	364,486 (2.62)	1 (Reference)		1 (Reference)	
Yes	636/22,483	3,053,921 (1.90)	1.39 (1.06–1.84)	0.019	1.62 (1.22–2.15)	< 0.001
Per 1 PHQ-9 score increase	NA	NA	1.03 (1.01–1.05)	0.002	1.04 (1.02–1.06)	< 0.001
Unfavorable SDoH						
Low burden	203/10,498	1,232,854 (1.27)	1 (Reference)		1 (Reference)	
High burden	514/14,229	2,185,554 (2.83)	2.36 (1.91–2.92)	< 0.001	2.09 (1.70–2.57)	< 0.001
Per 1 unfavorable SDoH increase	NA	NA	1.25 (1.20–1.30)	< 0.001	1.28 (1.20–1.35)	< 0.001
**Cancer mortality**						
Depression						
No	54/2,244	234,311 (1.69)	1 (Reference)		1 (Reference)	
Yes	552/22,483	2,850,187 (1.78)	0.96 (0.68–1.36)	0.800	1.02 (0.73–1.44)	0.900
Per 1 PHQ-9 score increase	NA	NA	1.01 (0.98–1.03)	0.600	1.02 (0.99–1.04)	0.300
Unfavorable SDoH						
Low burden	214/10,498	1,427,608 (1.47)	1 (Reference)		1 (Reference)	
High burden	392/14,229	1,656,890 (2.15)	1.54 (1.27–1.87)	< 0.001	1.51 (1.24–1.85)	< 0.001
Per 1 unfavorable SDoH increase	NA	NA	1.13 (1.07–1.18)	< 0.001	1.13 (1.05–1.21)	< 0.001

Model 1 was unadjusted. Model 2 was adjusted for age, sex, race/ethnicity, cardiovascular diseases, cancer, drinking status, smoking status, body mass index, sleep, physical activity, hypertension, diabetes mellitus, dyslipidemia

Abbreviations: SDoH: social determinants of health; HR: hazard ratio; CI: confidence interval; CVD: cardiovascular disease

The mediation analyses showed that SDoH played a significant mediating role, accounting for 32.4% (95% CI: 23.5–49.0) of the association between depression and all-cause mortality, as well as 28.3% (95% CI: 16.5–55.0) of the association between depression and CVD mortality. After including SDoH in the multivariate models, the HRs for the association between depression and both all-cause and CVD mortality risks were attenuated but remained significant compared to models without adjusting for SDoH (Table 3). The association of depression with mortality by levels of SDoH is presented in Supplementary Figure S2. These results suggested that the adverse effect of depression on mortality remained unchanged across participants with or without a low burden of unfavorable SDoH.

**Table 3:** Mediation of social determinants of health on associations between depression and all-cause and cause-specific mortality

Outcomes	HR (95%CI)		Mediation proportion (95%CI)
	Unadjusted for SDoH	Adjusted for SDoH	
**All-cause mortality**			
Depression			
Yes	1 (Reference)	1 (Reference)	
No	0.67 (0.58–0.79)	0.78 (0.67–0.92)	32.4 (23.5–49.0)
**CVD mortality**			
Depression			
Yes	1 (Reference)	1 (Reference)	
No	0.62 (0.47–0.82)	0.72 (0.54–0.96)	28.3 (16.5–55.0)
**Cancer mortality**			
Depression			
Yes	1 (Reference)	1 (Reference)	
No	0.98 (0.69–1.38)	1.08 (0.76–1.54)	29.0 (-1290.7-349.0)

Multivariate models were adjusted for age, sex, race/ethnicity, cardiovascular diseases, cancer, drinking status, smoking status, body mass index, sleep, physical activity, hypertension, diabetes mellitus, dyslipidemia

Abbreviations: SDoH: social determinants of health; HR: hazard ratio; CI: confidence interval; CVD: cardiovascular diseases

### Interaction and joint association of depression and SDoH with mortality

The stratified subgroup analysis based on depression status showed that regardless of depression, a low burden of unfavorable SDoH was associated with a decreased risk of all-cause mortality compared to a high burden of unfavorable SDoH. Among depressed participants, a low burden of unfavorable SDoH significantly decreased the all-cause mortality risk (HR, 0.58; 95% CI: 0.36–0.92). A low burden of unfavorable SDoH was also associated with lower risks of CVD and cancer mortality in those without depression (Supplementary Figure S3). However, there were no significant additive and multiplicative interactions between depression and SDoH on all-cause mortality (Additive: RERI: 0.18; 95% CI: -0.15-0.49; AP: 0.16, 95% CI: -0.19-0.52; SI: 0.81; 95% CI: 0.52–1.24; Multiplicative: HR: 1.16; 95% CI: 0.56–1.32), as well as CVD mortality and cancer mortality (Table 4).

**Table 4:** Additive and multiplicative interactions of depression and social determinants of health for all-cause and cause-specific mortality

Outcomes	Additive interaction			Multiplicative interaction	
	RERI	AP	SI	HR (95%CI)	*P* for interaction
All-cause mortality	0.18 (-0.15-0.49)	0.16 (-0.19-0.52)	0.81 (0.52–1.24)	1.16 (0.56–1.32)	0.489
CVD mortality	0.17 (-0.42-0.76)	0.18 (-0.52-0.88)	0.82 (0.38–1.74)	1.18 (0.36–1.98)	0.692
Cancer mortality	0.31 (-0.24-0.87)	0.31 (-0.41-1.02)	0.48 (0.11–2.18)	1.05 (0.37–2.45)	0.913

All models were adjusted for age, sex, race/ethnicity, cardiovascular disease, cancer, drinking status, smoking status, body mass index, sleep, physical activity, hypertension, diabetes mellitus, dyslipidemia

Abbreviations: SDoH: social determinants of health; CI: confidence interval; CVD: cardiovascular disease; RERI: relative excess risk due to interaction; AP: attributable proportion; SI: synergy index

**Fig. 1:**
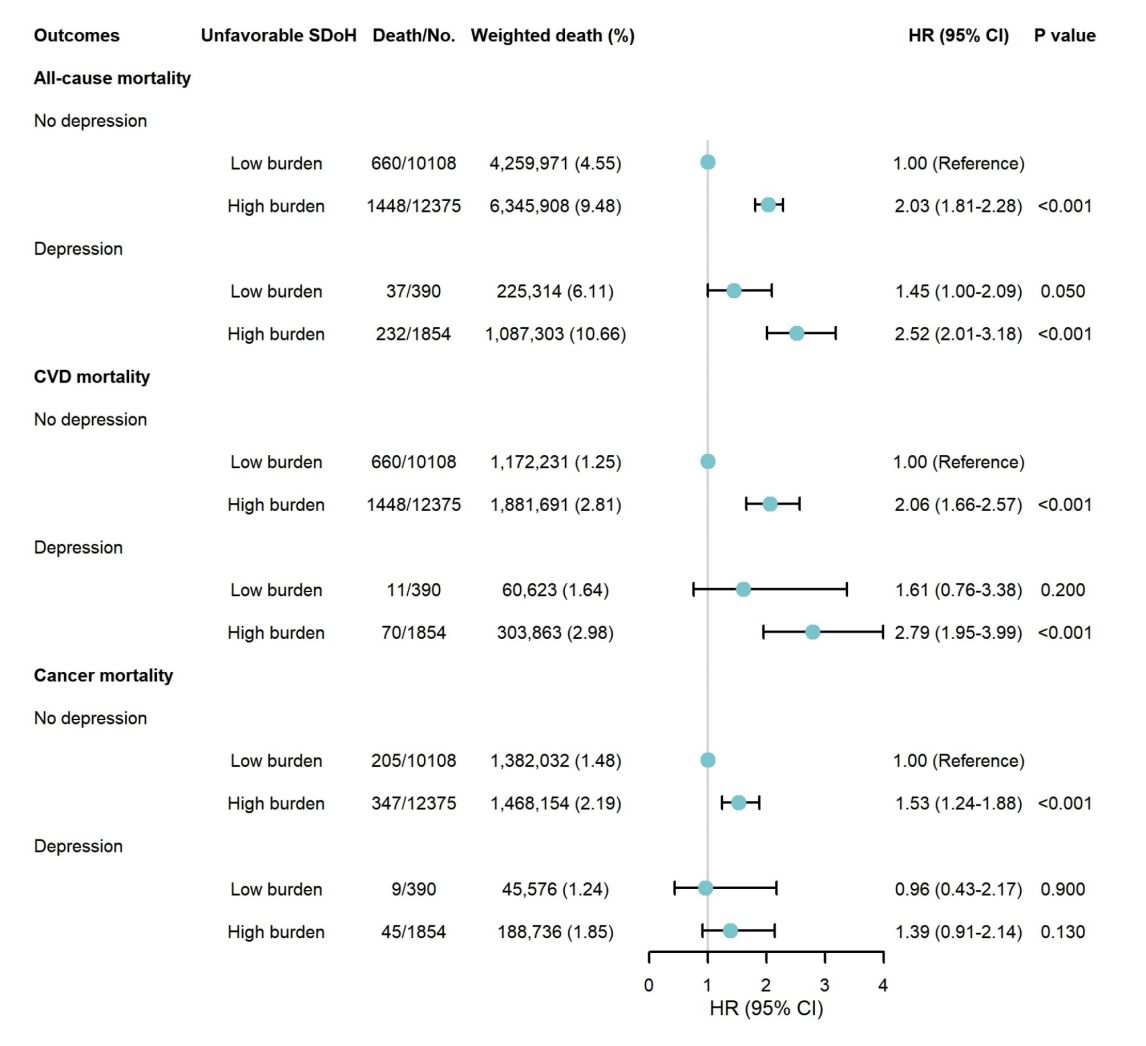
Joint associations of depression and social determinants of health with all-cause and cause-specific mortality. In this forest plot showing joint associations of depression and SDoH with mortality, the blue dots display HRs and the black horizontal lines through the dots display 95% CIs. All models were adjusted for age, sex, race/ethnicity, cardiovascular diseases, cancer, drinking status, smoking status, body mass index, sleep, physical activity, hypertension, diabetes mellitus, dyslipidemia. Abbreviations: SDoH: social determinants of health; HR: hazard ratio; CI: confidence interval; CVD: cardiovascular disease

Approximately 5.85% of participants with a high burden of unfavorable SDoH were also found to have depression, while 53.66% exhibited both no depression and a low burden of unfavorable SDoH (Supplementary Table S2). The KM curves showed that participants with both depression and a high burden of unfavorable SDoH showed the highest cumulative event rates of all-cause and CVD mortality (Supplementary Figure S4). In the joint analyses, the highest risks of all-cause and CVD mortality occurred among participants with depression and a high burden of unfavorable SDoH. Specifically, compared with those with no depression and a low burden of unfavorable SDoH, the HRs for all-cause and CVD mortality in the group with depression and a high burden of unfavorable SDoH were 2.52 (95% CI: 2.01–3.18) and 2.79 (95% CI: 1.95–3.99), respectively, while the risk of cancer mortality was not significant (Fig. 1).

### Sensitivity and subgroup analyses

Sensitivity analyses after excluding participants who died within the first 2 years of follow-up and those with CVD or cancer at baseline were shown in Supplementary Table S3 and Table S4. The joint association of depression and SDoH with risks of all-cause, CVD, and cancer mortality were roughly the same as before. In subgroup analyses, the joint associations of depression and SDoH with mortality remained consistent across different age, sex, race, CVD, and cancer subgroups (All P for interaction > 0.05) (Supplementary Table S5).

## Discussion

Using a nationally representative sample of U.S. adults, this prospective cohort study is the first to investigate the mediating, interacting, and joint effects of depression and SDoH in relation to mortality. There are 3 main findings. First, depression was independently associated with increased risks of all-cause and CVD mortality, with SDoH mediating 32.4% and 28.3% of the association between depression and all-cause and CVD mortality, respectively. Second, a low burden of unfavorable SDoH attenuated the risk of all-cause mortality due to depression, while there was no significant interaction between depression and SDoH. Third, there were joint effects of depression and SDoH on all-cause and CVD mortality. Individuals with both depression and a high burden of unfavorable SDoH had the highest risks of all-cause and CVD mortality.

Our results are consistent with previous evidence demonstrating the well-established independent association between depression or SDoH and elevated mortality risks. A meta-analysis of 293 studies with 1,813,733 participants from 35 countries showed that depression was associated with a 52% increased risk of all-cause mortality [5]. Increasing evidence suggests the role of SDoH as upstream drivers of observed disparities in mortality [31]. Recently, a cross-sectional study reported that cumulative unfavorable SDoH was associated with an increased risk of depression [17]. Previous research has also shown that the relationship between depression and incident CVD and mortality risks is particularly pronounced in low SES groups [20, 22]. Thus, it is reasonable to hypothesize that adverse outcomes in depressed individuals may be partially driven by their unfavorable SDoH. In the present study, the positive association between depression and mortality was attenuated when adjusting for SDoH, suggesting that SDoH may serve as a mediator between this association. Mediation analyses further confirmed this by indicating that SDoH played a modest mediating role in the association between depression and all-cause and CVD mortality. However, comparing this finding with previous studies on the joint effects of depression and SES on adverse clinical outcomes is difficult because mediating effects have not been assessed previously [21, 22], while the identification of SDoH as a partial mediator in the depression-mortality risk relationship suggests the possibly of addressing unfavorable SDoH to attenuate adverse effects of depression on mortality.

Furthermore, our study examined the interaction of depression and SDoH with mortality, which has not been explored in previous studies. Contrary to our hypothesis, there was no significant interaction between depression and SDoH on mortality risk. In essence, while both depression and unfavorable SDoH independently contribute to mortality risk, their joint effect did not appear to exceed the sum or product of their individual effects. The effects of multiple factors related to mortality may moderate the interaction between depression and SDoH, potentially explaining the lack of interaction. Additionally, depression and SDoH may affect mortality risk through different pathways. Future studies need to further elucidate the complex relationship of depression and SDoH with mortality and explore the underlying mechanisms. Although the interaction was not significant, our results showed that the low burden of unfavorable SDoH reduced the detrimental effects of depression on all-cause mortality risk through a stratified approach, suggesting that public health efforts targeting adverse SDoH may mitigate the mortality burden in depressed individuals.

It is well known that depression and SDoH are independent risk factors for mortality, however, the joint effect of these two risk factors on mortality remains unclear. Notably, we, for the first time, investigated the joint associations of depression and SDoH with mortality. Our findings revealed that individuals with depression and a high burden of unfavorable SDoH exhibited approximately 2.0-3.0-fold higher risks of all-cause and CVD mortality than those without either risk factor. Sensitivity and subgroup analyses consistently yielded robust results. This joint analysis enabled us to explore the individual and joint effects of depression and SDoH on mortality outcomes, thereby enhancing our understanding of survival outcomes among individuals with varying statuses of depression and SDoH. Our results align with previous studies, which demonstrated that individuals with both a low SES (measured by income, education, and occupation) and depression faced the highest risk of developing acute myocardial infarction and stroke compared to those with a high SES without depression from a population-based study of 2.7 million Korean adults [21]. Another prospective cohort study involving 466,239 UK Biobank participants reported that individuals with depression and a low SES (including education, income, deprivation) were at particularly high risk of major cardiovascular events [22]. Lazzarino et al. also found that a combination of psychological distress and low SES (measured by occupation) generated a 2.5-fold increase in all-cause mortality and a 2.0-fold increase in coronary heart disease and stroke mortality [32, 33]. However, such studies only evaluated a single determinant or SES that reflects certain domains of SDoH, yet we examined the joint effects of depression and cumulative all 5 domains of SDoH on mortality. These findings emphasize the importance of considering the role of SDoH alongside depression to mitigate mortality risks.

The mechanisms of the joint effects are potentially explained by psychosocial and biological mechanisms. Individuals with a high burden of unfavorable SDoH are exposed to more health adversity while having fewer strategies and healthcare support networks together with unhealthy behaviors and weak economic resources to deal with health-related problems [16], which contribute to being more susceptible to the deleterious effect of depression on survival. Additionally, unfavorable SDoH and depression are both associated with mortality through stimulation of stress hormone production, inflammation, and metabolic disorders [33]. Several studies have linked low SES to the body’s stress response via the hypothalamic-pituitary-adrenal axis and sympathetic nervous system [34, 35], suggesting that unfavorable SDoH may be considered psychosocial stressors with physiological implications. Thus, unfavorable SDoH not only contribute to disparities in access, treatment, and healthcare but also increases the risk of mortality through the stress pathophysiology related to depression.

Strengths of this study include the use of a large, nationally representative sample of general U.S. adults, a population-based design, and adjustment for multiple covariates. Furthermore, this study provides novel insights into the relationship between depression, SDoH, and mortality, which have practical implications for considering depression and SDoH jointly in clinical practice and public health. In clinical practice, our findings suggest that coexistence of depression and a high burden of unfavorable SDoH is at particularly high risks for mortality. To reduce mortality risk in these vulnerable and high-risk populations, routine screening for depression by using PHQ-9 and collection of SDoH by sociodemographic survey in clinics could help physicians and medical professionals identify these vulnerable individuals promptly, enabling more targeted and tailored preventive interventions. For public health, our findings may help guide the development of comprehensive public health policy strategies for depression screening and addressing adverse SDoH. These efforts should encompass strategies to establish multilevel partnerships between the healthcare system and community clinics, foster environments conducive to mental health, and promote access to public health resources. Our results also support the integration of SDoH and depression assessments into healthcare systems, resource allocation, and investment funding to develop strategies aimed at eliminating these risk factors for mortality prevention.

Nevertheless, several limitations should be acknowledged. Thus, our findings need to be interpreted with caution. First, both depression and SDoH were measured by self-reported, and recall and reporting biases may bias results towards the null, thereby underestimating the true magnitude of the joint association. Second, although our main analysis of adjusting multiple covariates and sensitivity analyses generated robust results, the possibility of reverse causation and residual confounding, such as many other diseases or medications, cannot be fully eliminated, which may have biased results towards the null. Third, NHANES used PHQ-9 rather than clinical criteria to diagnose depression, making it susceptible to information bias. However, PHQ-9 is a validated and accepted screening tool for depression. Fourth, some important SDoH domains were not widely available in NHANES, including experienced racism, discrimination, and social support. Thus, our findings are tentative pending replication in larger prospective cohort studies with good measures of depression and all domains of SDoH and in controlled trials of intervention on both risk factors. Fifth, the relatively small sample size in some joint groups (e.g., the depression-low burden of unfavorable SDoH combination) may have compromised the statistical power to detect differences in risk. Additionally, depression and SDoH were assessed only once at baseline, which limited the possibility of capturing long-term SDoH trajectories as well as dynamic changes in depressive status over time. Future studies with repeated measurements are preferred. Lastly, our findings may only be well representative of U.S. population, and further cohort studies are warranted to verify the generalizability of the findings to other populations.

## Conclusion

In a nationally representative sample of U.S. adults, the coexistence of depression and a high burden of unfavorable SDoH was significantly associated with increased risks of all-cause mortality and CVD mortality. These findings suggest integrating both depression and SDoH jointly into clinical practice and public health strategies to improve survival. Future larger prospective cohort studies and clinical trials are required to validate our findings.

## Electronic supplementary material

Below is the link to the electronic supplementary material.

Supplementary Material 1

## Data Availability

The datasets generated and analyzed in the current study are available on the National Health and Nutrition Examination Survey (NHANES) website: https://wwwn.cdc.gov/nchs/nhanes/.

## Abbreviations

AP: Attributable proportion
BMI: Body mass index
CI: Confidence interval
CVD: Cardiovascular disease
DM: Diabetes mellitus
HR: Hazard ratio
KM: Kaplan-Meier
NHANES: National Health and Nutrition Examination Survey
PHQ-9: Patient Health Questionnaire-9
PA: Physical activity
RERI: Relative excess risk due to interaction
SDoH: Social determinants of health
SES: Socioeconomic status
SE: Standard error
SI: Synergy index
WHO: World Health Organization
